# Statins for the prevention of cardiovascular events associated with avian influenza: the COVID-19 pandemic as a reference

**DOI:** 10.1080/07853890.2024.2390166

**Published:** 2024-08-17

**Authors:** Alpo Vuorio, Bruce Budowle, Frederick Raal, Petri T. Kovanen

**Affiliations:** a Mehiläinen, Airport Health Center, Vantaa, Finland; b Department of Forensic Medicine, University of Helsinki, Helsinki, Finland; c Faculty of Health Sciences, University of Witwatersrand, Johannesburg, South Africa; d Wihuri Research Institute, Helsinki, Finland

**Keywords:** Avian influenza, cardiovascular, COVID-19, endothelial dysfunction, H5N1 influenza virus, pandemic, statins

## Abstract

There is growing concern that the severe respiratory disease in birds (avian influenza or ‘bird flu’) caused by the H5N1 influenza virus, might potentially spread more widely to humans and cause a pandemic. Here we discuss clinical issues related to human infections by the highly pathogenic H5N1 subtype of the avian influenza A virus and make a clinical comparison with recent information obtained from studies of SARS-CoV-2 infection. Firstly, we consider the potential increase in cardiovascular events in humans infected with the H5N1 virus. Like SARS-CoV-2 infection, H5N1 infection may result in endothelial dysfunction and the associated procoagulant and prothrombotic state, and *via* this mechanism, the infection can potentially increase cardiovascular morbidity, especially in vulnerable individuals with pre-existing cardiovascular disease. Secondly, we discuss the potential beneficial role of statin use, both in the prophylaxis and the treatment of individuals with influenza A(H5N1), as was found favorable for the treatment of COVID-19 caused by SARS-CoV-2 infection.

## KEY MESSAGES

There is a concern that avian influenza caused by the highly pathogenic avian influenza A(H5N1) virus might potentially spread more widely to humans and result in a pandemic

H5N1 infection may result in endothelial dysfunction and via this mechanism, it can potentially increase cardiovascular morbidity and mortality as has occurred with SARS-CoV-2 infection.

There is a potential advantage of the use of statins to reduce cardiovascular morbidity and mortality in patients with avian influenza A(H5N1), as has been found in patients suffering from COVID-19.

## Graphical Abstract

**Figure:**
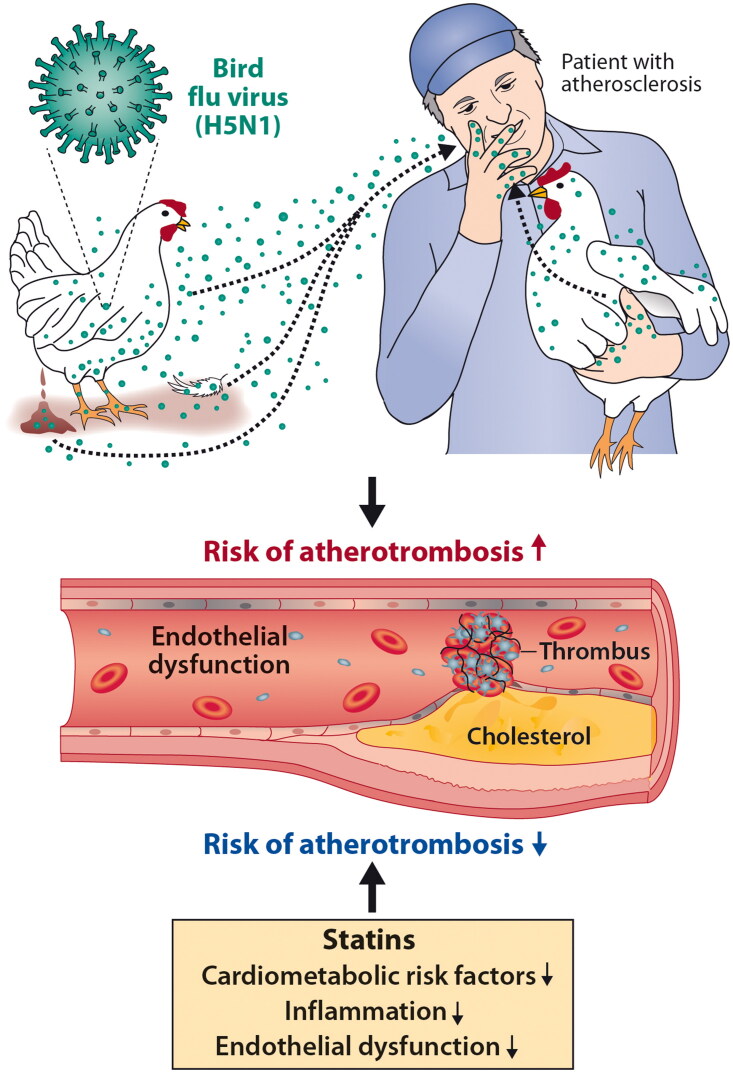

## Introduction

There is a concern that avian influenza caused by the highly pathogenic influenza (A)H5N1 virus might potentially spread more widely to humans and result in a further pandemic [1]. Indeed, H5N1 infection may potentially increase cardiovascular morbidity as has been seen with COVID-19 [2]. The advantage of the use of statins for the reduction of cardiovascular morbidity and mortality caused by the H5N1 virus, and possibly by other pathogenic H5N(x) subtypes, remains uncertain, but may be beneficial as has been observed in patients with SARS-CoV-2 infection [3, 4]. In this article, we discuss clinical issues related to H5N1 virus infection and compare them with novel information obtained from ongoing studies with SARS-CoV-2 infection.

The known history of avian influenza traces back to Northern Italy in the year 1878 [5]. In that year an infectious disease with high mortality in poultry was described. The first major human influenza pandemic, i.e. the Spanish Flu, occurred in 1918 [6]. This pandemic caused by the highly pathogenic H1N1 subtype of the avian influenza A virus, resulted in approximately 50 million deaths worldwide. Honigsbaum [7] noted that in the pre-COVID-19 years, Spanish influenza, the mother of all pandemics, ‘had somehow been forgotten or excised from public memory’. It is conceivable that the same variant or derivatives of the 1918 avian influenza A virus are still circulating in both humans and animals [8] and may well reignite resulting in another influenza pandemic.

The world is just recovering from the COVID-19 pandemic that, according to the most recent validated scientific evidence, was transmitted to humans from bats [9, 10]. Unfortunately, the respite may be short-lived, as recent news from Ecuador and Cambodia alludes to a possible outbreak of H5N1 influenza A, which could result in another pandemic. On 9 January 2023, a nine-year-old girl living in a rural area in Ecuador who had had contact with poultry was reported to have influenza tested positive for the influenza A(H5N1) virus [11]. The story continues, as two human influenza patients in Cambodia were also infected with the H5N1 virus [12]. These H5N1 viruses were phylogenetically different from those currently circulating in poultry in the United States and other countries. Despite no known cases of human-to-human transmission of the avian influenza A(H5N1) virus, there is growing concern that to date no vaccine is available for prophylaxis [13]. On the other hand, there are four FDA-approved influenza antiviral drugs for use against recently circulating other influenza virus variants [14].

In this commentary, we discuss two clinical issues that relate to a potential human influenza pandemic due to exposure to the avian influenza A(H5N1) (bird flu) virus (see graphical abstract). Firstly, there is likely to be an increase in cardiovascular events in humans infected with the H5N1 virus. Like SARS-CoV-2 infection, influenza A(H5N1) infection may result in endothelial dysfunction and thereby potentially increase cardiovascular morbidity and mortality, especially in vulnerable individuals with pre-existing cardiovascular disease [15–17]. Secondly, we propose that there may be a role for statins in the treatment or prophylaxis of influenza A(H5N1). While statins have been used to treat COVID-19 comorbidities successfully, there is less knowledge or experience regarding the potential beneficial effects of statins among H5N1-infected individuals [18, 19].

## Discussion

Although the information on the deleterious effects of influenza A(H5N1) on the endothelium is still uncertain, compared to the well-documented adverse effects on the endothelium of SARS-CoV-2 virus infection, both animal studies and human clinical studies are available to provide some insight with relevance to the understanding of the increased risk of cardiovascular events associated with these infections. Armstrong et al. [15] suggested that during an H5N1 infection, in addition to the body’s immune response, endothelial dysfunction with resultant loss of the endothelial barrier function and consequent microvascular leakage will ensue. The authors highlighted that inflammation, permeability, and thrombosis also pertain to the endothelial cells of the vessels in the microvascular trees. Similar responses have been observed in the pathogenesis of a SARS-CoV-2 infection [17]. Moreover, like in COVID-19, a cytokine storm accompanies the often fatal avian influenza A(H5N1) disease, as it affects the endothelium, and causes its systemic dysfunction [20, 21]. Tumor necrosis factor (TNF) is one of the key cytokines implicated in the pathogenesis of an avian influenza A(H5N1) infection [22]. Also, this observation is similar to what was found in COVID-19 in which the magnitude of the increase in the serum concentration of TNF predicts the prognosis of the disease [23]. Analogously to what was seen in SARS-CoV-2 infection, in influenza A(H5N1) virus infection TNF, IL-6, and IL-1β have been shown to upregulate trypsin, an enzyme capable of degrading the endothelial tight junction protein zonula occludens-1, and thereby to trigger vascular hyperpermeability [24, 25]. In influenza A(H5N1) virus infection endothelial dysfunction also involves the microvascular beds as observed in SARS-CoV-2 infection [26, 27]. It is therefore highly feasible that in these two diseases, the resulting endothelial damage is caused by both proinflammatory cytokines and a direct viral infection of the endothelial cells themselves.

An additional endothelium-related pathogenic mechanism in avian influenza A(H5N1) is increased thrombogenesis. Animal studies have documented that H5N1 infection causes coagulopathy [28, 29]. In humans, a clinical study of 119 hospitalized avian influenza A(H1N1) patients reported that 5.9% of the patients had a thrombotic vascular event [30]. In this study, the vascular events were defined as either thrombotic or embolic events occurring either in the venous or arterial circulation during hospitalization. This incidence can be compared with the European network cohort study of hospitalized COVID-19 patients [31] in which the cumulative 90-day incidence of venous thromboembolism was up to 4.5%, with an incidence of arterial thromboembolism of 3.1%. In a Chinese study of 321 hospitalized patients with avian influenza H7N9, 203 cases (63%) showed evidence of cardiac injury [32]. The cardiac injury was studied further using electrocardiograms, laboratory tests (troponin and creatine kinase-myocardial brand), and echocardiography. The authors found that cardiac injury was independently associated with the risk of mortality during hospitalization. In a study of 37 patients admitted to the intensive care unit with severe pandemic influenza (H1N1) 2009 virus infection, 17 (46%) had evidence of myocardial injury, and of these 17 patients, 14 (82%) had myocarditis [33]. In the meta-analysis of hospitalized COVID-19 patients, the prevalence of myocardial injury ranged from 9.2 to 51%, with a mean prevalence of 27.2% [34]. Whether the myocardial damage has been ischemic, i.e. caused by endothelial damage and ensuing thrombus formation in the cardiac microvascular bed, or whether it resulted from a direct inflammatory or viral damage of the myocardial cells, or has resulted from both, remains uncertain.

As noted above, no H5N1 vaccines are currently available to the general population [13]. While antiviral drugs can be used to treat H5N1 avian influenza, the development of drug resistance may hinder their effectiveness. To increase the drug arsenal in patients showing delayed or poor recovery, statins could serve as an adjunctive beneficial class of drugs. In a controlled mouse study, in which the effects of several types of statins on the highly pathogenic avian influenza H5N1 and on seasonal and H1N1pdm09 virus infections were investigated in BALB/c mice, statin treatment did not show any significant efficacy in improving the prognosis of the disease [35]. In addition, in a study with BALB/c mice, simvastatin did not improve the effectiveness of the antiviral medication oseltamivir [36]. However, in a meta-analysis of 14,997 influenza-vaccinated and unvaccinated patients, statin use was associated with a reduced prevalence of influenza H1N1 infection with an odds ratio of 0.54 (95% confidence interval: 0.33, 0.87) and with reduced mortality with an odds ratio of 0.64 [0.46, 0.88] [37]. In COVID-19 infection, the beneficial effects of statins are more substantiated [18, 38, 39].

## Conclusions

There is a potential risk of human-to-human transmission of avian influenza A(H5N1) virus. The virus has already been detected in minks and cows, which may serve as an intermediate host and pose further risk to human infections [40–44]. In fact, the current situation is reminiscent of the transmission of SARS-CoV-2 from minks to humans [41].

If another H5N1 pandemic were to occur, the number of cardiovascular cases related to this infection as well as mortality from cardiovascular complications would inevitably rise, which in turn would create additional burdens on the healthcare system. As discussed above, there is some evidence that statin therapy could potentially be beneficial for patients with H5N1 infection [33]. Yet, based on current but very limited information, there is no reason to discontinue statin treatment when one has influenza. Rather, right now when we live in fear of a potential further viral pandemic, effective statin medication which has been prescribed for the prevention of cardiovascular events should be continued prophylactically. It is feasible, that statins, which are cardioprotective and have anti-inflammatory and immunomodulatory effects, have the potential to reduce anticipated cardiovascular comorbidities related to H5N1 infection. However, more data are urgently needed, particularly since it is conceivable that highly pathogenic avian influenza A(H5N1) virus variants with pandemic potential may emerge.

## Data Availability

Data sharing is not applicable to this article as no new data were created or analyzed in this study.
